# Cardiac magnetic resonance reveals biventricular impairment in Cushing’s syndrome: a multicentre case-control study

**DOI:** 10.1007/s12020-024-03856-7

**Published:** 2024-05-22

**Authors:** Tiziana Feola, Alessia Cozzolino, Dario De Alcubierre, Riccardo Pofi, Nicola Galea, Carlo Catalano, Chiara Simeoli, Nicola Di Paola, Federica Campolo, Rosario Pivonello, Andrea M. Isidori, Elisa Giannetta

**Affiliations:** 1 Department of Experimental Medicine, Sapienza University of Rome, Rome, Italy; 2 Neuroendocrinology, Neuromed Institute, IRCCS, Pozzilli, Italy; 3 Oxford Centre for Diabetes, Endocrinology, and Metabolism, Churchill Hospital, Oxford University Hospitals, NHS Trust, Oxford, UK; 4 Department of Radiological Sciences, Oncology and Pathology, Sapienza University of Rome, Rome, Italy; 5 Dipartimento di Medicina Clinica e Chirurgia, Università Federico II di Napoli, Naples, Italy; 6 Centre for Rare Diseases (ENDO-ERN accredited), Policlinico Umberto I, Rome, Italy

**Keywords:** Cushing’s syndrome, Hypercortisolism, Glucocorticoid, Cardiomyopathy, Heart, Cardiac magnetic resonance

## Abstract

**Purpose:**

Cushing’s syndrome (CS) is associated with severe cardiovascular (CV) morbidity and mortality. Cardiac magnetic resonance (CMR) is the non-invasive gold standard for assessing cardiac structure and function; however, few CMR studies explore cardiac remodeling in patients exposed to chronic glucocorticoid (GC) excess. We aimed to describe the CMR features directly attributable to previous GC exposure in patients with cured or treated endogenous CS.

**Methods:**

This was a prospective, multicentre, case-control study enrolling consecutive patients with cured or treated CS and patients harboring non-functioning adrenal incidentalomas (NFAI), comparable in terms of sex, age, CV risk factors, and BMI. All patients were in stable condition and had a minimum 24-month follow-up.

**Results:**

Sixteen patients with CS and 15 NFAI were enrolled. Indexed left ventricle (LV) end-systolic volume and LV mass were higher in patients with CS (*p* = *0.027; p* = *0.013*); similarly, indexed right ventricle (RV) end-diastolic and end-systolic volumes were higher in patients with CS compared to NFAI (*p* = *0.035; p* = *0.006*). Morphological alterations also affected cardiac function, as LV and RV ejection fractions decreased in patients with CS (*p* = *0.056; p* = *0.044*). CMR features were independent of metabolic status or other CV risk factors, with fasting glucose significantly lower in CS remission than NFAI (*p* < 0.001) and no differences in lipid levels or blood pressure.

**Conclusion:**

CS is associated with biventricular cardiac structural and functional impairment at CMR, likely attributable to chronic exposure to cortisol excess independently of known traditional risk factors.

## Introduction

Cushing’s syndrome (CS), or chronic hypercortisolism, is associated with increased mortality mostly due to cardiovascular disease [1, 2], with infectious diseases and coexisting comorbidities also playing a role [1–9]. Older age at diagnosis, longer disease activity, uncontrolled hypertension, and diabetes mellitus are the main factors increasing mortality in CS [1, 2].

The higher cardiovascular risk in CS has traditionally been attributed to chronic hypertension, vascular atherosclerosis, and increased thromboembolism [2, 10–14], ultimately leading to an increased risk for myocardial infarction, cardiac failure, and stroke [2, 15].

Prompt and effective treatment of cortisol excess is crucial for reversing comorbidities and reducing the mortality risk associated with CS [1, 2]. However, concomitant treatment for cardiovascular comorbidities should also be provided to mitigate cardiovascular damage [1, 2, 16]. Despite improved treatment modalities, comorbidities can persist in a significant proportion of patients even after remission of CS [2, 17], suggesting that the consequence of prolonged exposure to glucocorticoid (GC) excess can produce irreversible alteration in cardiac structure.

Alterations in cardiac kinetics and structure include abnormal relaxation patterns (decreased systolic strain and impaired diastolic filling) and concentric left ventricle hypertrophy [2, 18–20], the latter being more severe in CS patients when compared to hypertensive controls [2, 20]. Increased myocardial fibrosis, caused by enhanced responsiveness to angiotensin II and activation of the mineralocorticoid receptor in response to cortisol excess, further complicates the scenario [2].

Albeit myocardial fibrosis and cardiac abnormalities might improve [21], cardiovascular alterations can persist for up to 5 years since remission of GC excess [2, 22, 23], underscoring the importance of prompt diagnosis and treatment, but also monitoring of increased risk.

Most studies rely on 2D echocardiography to characterize cardiac alterations in patients with CS [24]. Still, cardiac magnetic resonance (CMR) is now the established non-invasive gold standard method for measuring left ventricle (LV) volume, LV mass (LVM), and cardiac function due to its higher accuracy, reproducibility, and lower variability [25]. Few controlled studies assess cardiac dysfunction in CS patients by CMR, with preliminary data confirming the 2D-echocardiography observation of altered LV function and structure [18, 24, 26–29].

Therefore, our study aims to provide a detailed characterization of cardiac alterations in patients who have been exposed to chronic GC excess using a CMR-based approach and help clarify which are directly attributable to GC excess by matching the CS cohort with randomly selected adrenal patients with proven intact hypothalamic-pituitary-adrenal-axis, but similar traditional cardio-metabolic risk factors.

## Materials and methods

### Study design and population

We performed a prospective, multicentric, case-control study. From September 2014 to January 2020, consecutive adult (>18 years) patients diagnosed with CS as per current criteria [30] (either cured or with an active drug-treated disease) were recruited from the endocrinology outpatient clinics of the Department of Experimental Medicine at “Sapienza” University of Rome and the Department of Clinical Medicine and Surgery at “Federico II” University of Naples. Disease remission following surgery was defined by urinary free cortisol (UFC) levels per upper limit of normal (ULN) < 1.0 and by serum morning cortisol levels <50 nmol/L following overnight 1 mg dexamethasone suppression, in the absence of any cortisol-lowering treatment. Disease control under chronic medical therapy was defined by UFC xULN <1.0 [16, 31]. Patients with contraindications (or unwilling to undergo) to CMR were excluded from the study. The control group consisted of randomly selected patients with non-functioning adrenal incidentalomas (NFAI) diagnosed according to current criteria [32] undergoing follow-up imaging for the adrenal lesion, comparable with patients in terms of sex, age, BMI, and traditional cardiovascular risk factors. Sixteen patients with CS and 15 NFAI entered the study. All patients must have been in stable condition, including hormonal control, for at least 6 months before entering the study. All patients provided written informed consent after fully explaining the purpose and nature of all procedures used. The study was approved by the Ethical Committee of Policlinico Umberto I (ref. number 4245). The study has been performed according to the ethical standards of the 1964 Declaration of Helsinki and its later amendments. This study adhered to the Strengthening the Reporting of Observational Studies in Epidemiology (STROBE) guidelines for reporting.

### Study procedures

#### Clinical and laboratory assessment

All patients underwent an accurate medical history review, including drugs used, hormonal assessment at diagnosis (UFC xULN, serum cortisol after dexamethasone suppression test), comorbidities (hypertension, glucose metabolism impairment, dyslipidemia, obesity), and cardiovascular risk factors (e.g., smoking habit). Subsequently, they were submitted to physical examination with measurement of anthropometric parameters and vital signs. Blood sampling for the assessment of biochemistry and hormones was performed at the local laboratory of each participating center; to better standardize results about disease activity, UFC levels were normalized by the upper limit of normal of each center’s laboratory. Clinical and laboratory findings and the prevalence of cardiometabolic complications have been compared between patient groups (CS vs NFAI) and cured and drug-treated patients (cured CS vs controlled CS). All patients were followed up for a minimum of 24-month timeframe.

#### Cardiac evaluation

All subjects underwent cardiac evaluation with CMR imaging performed as previously described [33] with a 1.5-T clinical magnetic resonance imaging system (Avanto, Siemens, Healthcare Solutions, Erlangen, Germany). During the examination, an ECG device was used for cardiac gating, and all acquisitions were made in apnea at the end of inspiration. In all cases, CMR imaging was performed by the same radiologist expert in cardiac imaging (N.G.) using the same acquisition protocol. CMR was performed at study entry, but not earlier than 6 months from any previous severe acute disease, event, or procedure.

#### T1-mapping for the evaluation of fibrosis

The T1-mapping technique has been used to quantify the degree of myocardial fibrosis non-invasively. The measured extracellular volume fraction (ECV) is highly sensitive and indicates diffuse myocardial fibrosis [34]. T1-mapping is automatically calculated as the average of the intensity of the individual pixels with and without contrast medium in T1 and expressed in msec (CMR 42 SW). The calculation of the ECV has been performed using the mathematical formula using the hematocrit value [DR1 myocardium: (1/T1 myocardial-post) − (1/T1 myocardial-pre); DR1 blood: (1/T1 blood-post) − (1/T1 blood-pre); Myocardial partition coefficient (λ) = (DR1 myocardial/DR1 blood); ECV = (1 − hematocrit) × (λ)]. A cut-off of ECV > 30% was used to identify increased interstitial fibrosis [35].

CMR findings have been compared between patient groups (CS vs NFAI). Moreover, the comparison of cardiac parameters has also been performed in CS patients according to cardiometabolic comorbidities, disease status, and sex.

**Fig. 1:**
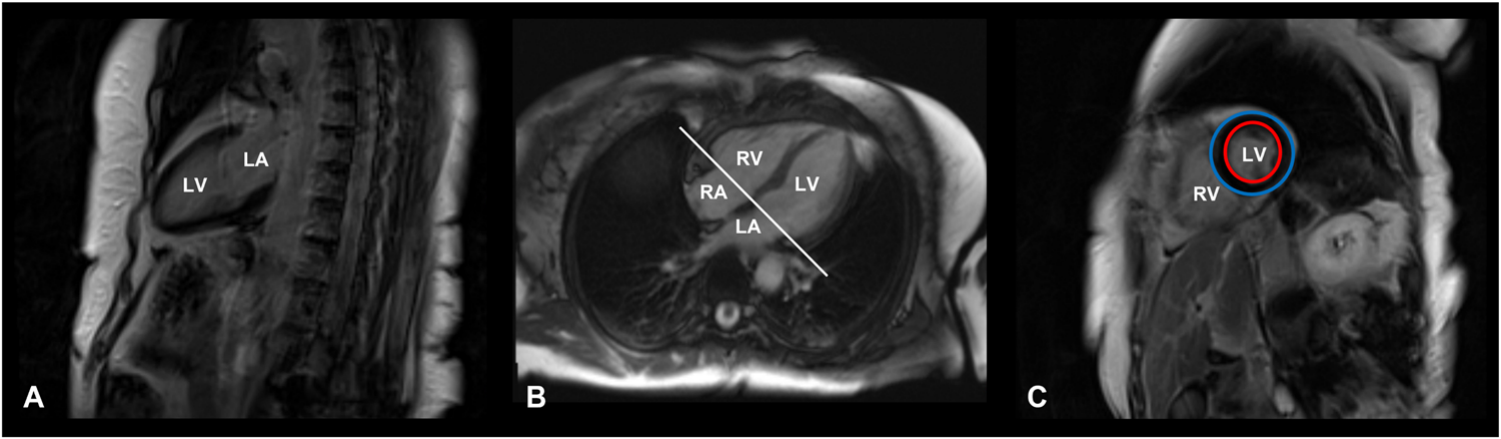
Cardiac magnetic resonance image acquisition protocol. T1-weighted, late gadolinium enhancement cardiac MR images of a 71-year-old female patient with Cushing’s disease, cured after successful neurosurgery. **A** Vertical long axis slice, coronal plane, two-chamber view. **B** Horizontal long axis slice, axial plane, four-chamber view. **C** Short-axis slice at the end of the diastole, sagittal plane. Red circle: endocardium; Blue circle: epicardium. LA left atrium, LV left ventricle, RA right atrium, RV right ventricle

The main steps of CMR image acquisition are shown in Fig. 1.

### Statistical analysis

Continuous variables are expressed as standard deviation (SD), median and 95% confidence interval (95%CI) as per data distribution, assessed through the Shapiro–Wilk test. Dichotomous variables are expressed as frequencies and percentages when relevant. According to variable distribution, the Student’s t-test or the non-parametric Mann–Whitney U test was performed to compare continuous variables between CS and NFAI and between cured and drug-controlled patients. Differences between groups regarding qualitative variables were evaluated by χ^2^ statistics. Bivariate correlations between numerical variables were analyzed using Pearson’s or Spearman’s correlation test, as appropriate. The statistical significance was set at *p* < 0.05. Statistical analyses were performed using SPSS 20.0 for MacOS (SPSS Inc.).

## Results

### Patient characteristics

The cohort characteristics are summarized in Table 1. Sixteen patients with CS (12 females, mean age 47 ± 12 years) and 15 with NFAI (7 females, mean age 55 ± 10 years) were enrolled during the study period. Twelve patients (75%) had been diagnosed with Cushing’s disease (CD), while four (25%) had ACTH-independent CS due to a cortisol-secreting adrenal adenoma.

**Table 1:** Baseline characteristics of patients with CS

Sex (M/F)	4/12
Age, years (mean ± SD)	47.5 ± 12
Disease status (cured/drug-treated)	11/5
Time from diagnosis, years (median, IQR)	3.0 (1.0–12.0)
CS origin (pituitary/adrenal)	12/4
Neurosurgery, *n* (%)	9 (75.0% of pituitary patients)
Hypocortisolism, *n* (%)	8 (50.0%)
Hypothyroidism, *n* (%)	5 (31.2%)
Hypogonadism, *n* (%)	2 (50.0% of males)
Menopause, *n* (%)	5 (41.7% of females)
Adrenal surgery, *n* (%)	4 (100.0% of adrenal patients)
Metyrapone, *n* (%)	2 (12.4%)
Cabergoline, *n* (%)	3 (18.7%)

*M* males, *F* females, *SD* standard deviation, *IQR* Interquartile Range, *n* number, *CS* Cushing’s syndrome

At enrollment, eleven (69%) patients were cured and five (31%) had drug-treated CD. Among patients with CD, nine had previously undergone pituitary surgery, seven (58%) were cured, and five (42%) presented with a biochemically persistent disease. In the latter group, 3 patients had adequate biochemical control under medical therapy, whereas 2 patients were not entirely on target, because of low compliance and intolerance to medical treatments.

All patients with a cortisol-secreting adrenal adenoma had undergone unilateral adrenalectomy and were cured at the time of enrollment.

Table 2 details comorbidities and their therapies for CS and NFAI patients.

**Table 2:** Biochemical and clinical parameters in CS patients and NFAI

	CS (*n* = 16)	NFAI (*n* = 15)	*p* value
Sex (M/F)	4/12	8/7	0.106
Age, years	47.5 ± 12.1	55.2 ± 9.7	0.062
BMI, kg/m^2^	28.9 (24.0–36.5)	26.5 (24.5–30.1)	0.830
Waist circumference, cm	99.0 (88.0–120.0)	101 (91.0–105.0)	0.898
Systolic BP, mmHg	118 (100-125)	120 (110–130)	0.626
Diastolic BP, mmHg	77 (70–85)	80 (70–80)	0.401
Heart rate, beats/min	72.6 ± 9.3	70.3 ± 8.5	0.481
Fasting plasma glucose, mmol/L	4.27 (4.00–4.50)	5.50 (4.61–6.88)	**<0.001**
HbA1c, %	5.5 ± 0.38	5.8 ± 0.48	0.161
Fasting insulin, µUI/mL	9.2 (6.1–17.5)	9.7 (7.4–17.0)	0.830
HOMA-IR	1.81 (1.16–3.54)	2.08 (1.49–5.10)	0.458
Triglycerides, mg/dL	104.0 (81.2–127.7)	120.0 (77.0–163.0)	0.401
Total cholesterol, mg/dL	199.5 ± 24.7	199.9 ± 41.6	0.976
HDL cholesterol, mg/dL	60.6 ± 12.1	57.6 ± 10.8	0.480
LDL cholesterol, mg/dL	118.0 (102.7–130.0)	105.0 (105.0–153.0)	0.446
Hypertension, *n* (%)	9 (56.2%)	6 (40.0%)	0.366
Obesity, *n* (%)	4 (25.0%)	4 (26.6%)	0.916
Diabetes, *n* (%)	1 (6.2%)	5 (33.3%)	0.056
IGT, *n* (%)	5 (31.2%)	3 (20.0%)	0.474
IFG, *n* (%)	0 (0.0%)	3 (20.0%)	0.060
Dyslipidemia, *n* (%)	7 (43.7%)	6 (40.0%)	0.833
Anti-hypertensive drugs, *n* (%)	9 (56.2%)	6 (40.0%)	0.366
Anti-diabetic drugs, *n* (%)	1 (6.25%)	4 (26.6%)	0.122
Lipid-lowering drugs, *n* (%)	5 (31.2%)	4 (26.6%)	0.779

Values are presented as mean ± SD or as median, Interquartile Range (IQR) according to the distribution of variables. Bold values indicate a *p* value lower than 0.05 as statistically significant.

*CS* Cushing’s syndrome, *NFAI* non-functioning adrenal incidentalomas, *n* number, *BMI* body mass index, *BP* blood pressure, *HOMA-IR* homeostatic model assessment for insulin resistance, *IGT* impaired glucose tolerance, *IFG* impaired fasting glucose

*P* values < 0.05 were considered statistically significant

### Biochemical and clinical evaluation

The main clinical and biochemical parameters are reported in Table 2. Sex, age, and BMI did not differ between the CS patients and NFAI. The two groups were similar concerning HbA1c, fasting insulin or homeostatic model assessment for insulin resistance, and lipid levels; fasting glucose levels were marginally lower in CS than in NFAI (*p* < 0.001). No differences were found in systolic and diastolic blood pressure, the prevalence of cardiometabolic complications or drugs (i.e., diagnosis of hypertension, dyslipidemia, obesity, and diabetes or prescriptions needed to control such comorbidities), suggesting that GC excess was resolved (or adequately controlled) at the time of enrollment for the great majority of patients.

### Subgroup analysis of cardiac parameters in patients with Cushing’s syndrome

A subgroup analysis was performed to assess possible differences in cardiac parameters when the CS cohort was stratified according to disease status and cardiometabolic comorbidities (i.e., between patients with or without hypertension, glucose metabolism impairment, dyslipidemia, obesity), smoking, sex, and disease status.

Firstly, pharmacologically treated CS exhibited higher systolic and diastolic blood pressure levels than cured CS (*p* = *0.027*), but no other differences were found regarding CMR parameters. Subgroup analysis according to the presence/absence of comorbidities revealed no significant effect on cardiac parameters, except for CS patients with impaired glucose tolerance, who showed a lower RV-EF compared with the remaining CS patients (*p* = *0.017*).

Analyzing sex differences, male CS patients displayed higher RV-EDVi (*p* = *0.035*) and RV-ESVi (*p* = *0.044*), as well as a trend toward higher LV-EDVi (*p* = *0.067*), LV-ESVi (*p* = *0.053*) and LVMi (*p* = *0.066*) compared to females. However, male CS patients only exhibited higher interventricular septum (IVS) thickness (*p* = *0.001*) when compared to male and female reference ranges for the general population, age and sex-matched [36].

### Comparison of cardiac parameters between patients and controls

**Fig. 2:**
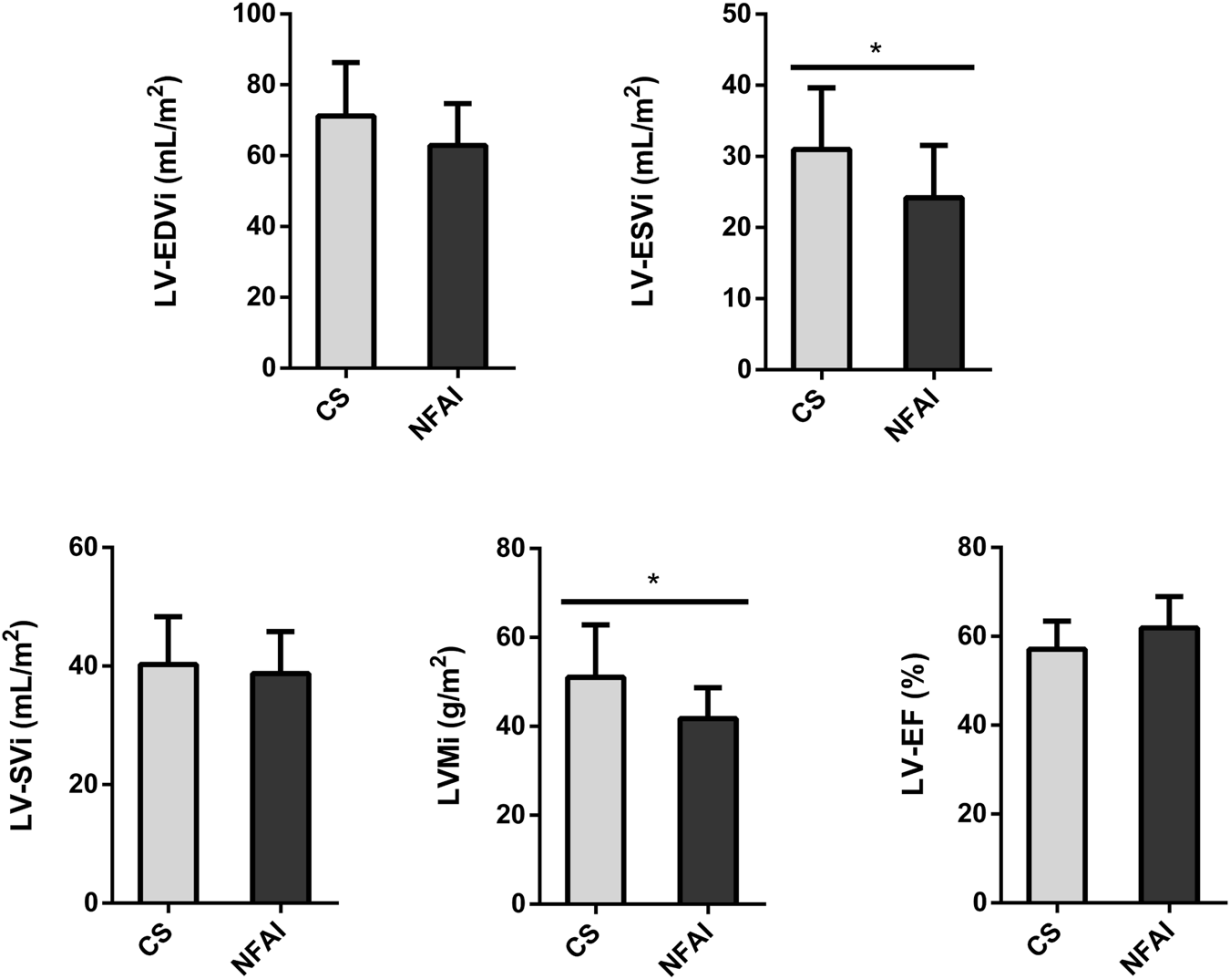
Comparative analysis of left ventricle parameters in Cushing’s syndrome and NFAI patients. Left ventricle morphological and functional cardiac parameters in patients with Cushing’s syndrome (gray bars) and patients with NFAI (black bars). Data are expressed as mean ± SD. *p < 0.05. CS Cushing’s syndrome, CNT NFAI, LV-EDVi Left Ventricle End-Diastolic Volume index, LV-ESVi Left Ventricle End-Systolic Volume index, LV-SVi Left Ventricle Stroke Volume index, LVMi Left Ventricular Mass index, LV-EF Left Ventricle Ejection Fraction

**Fig. 3:**
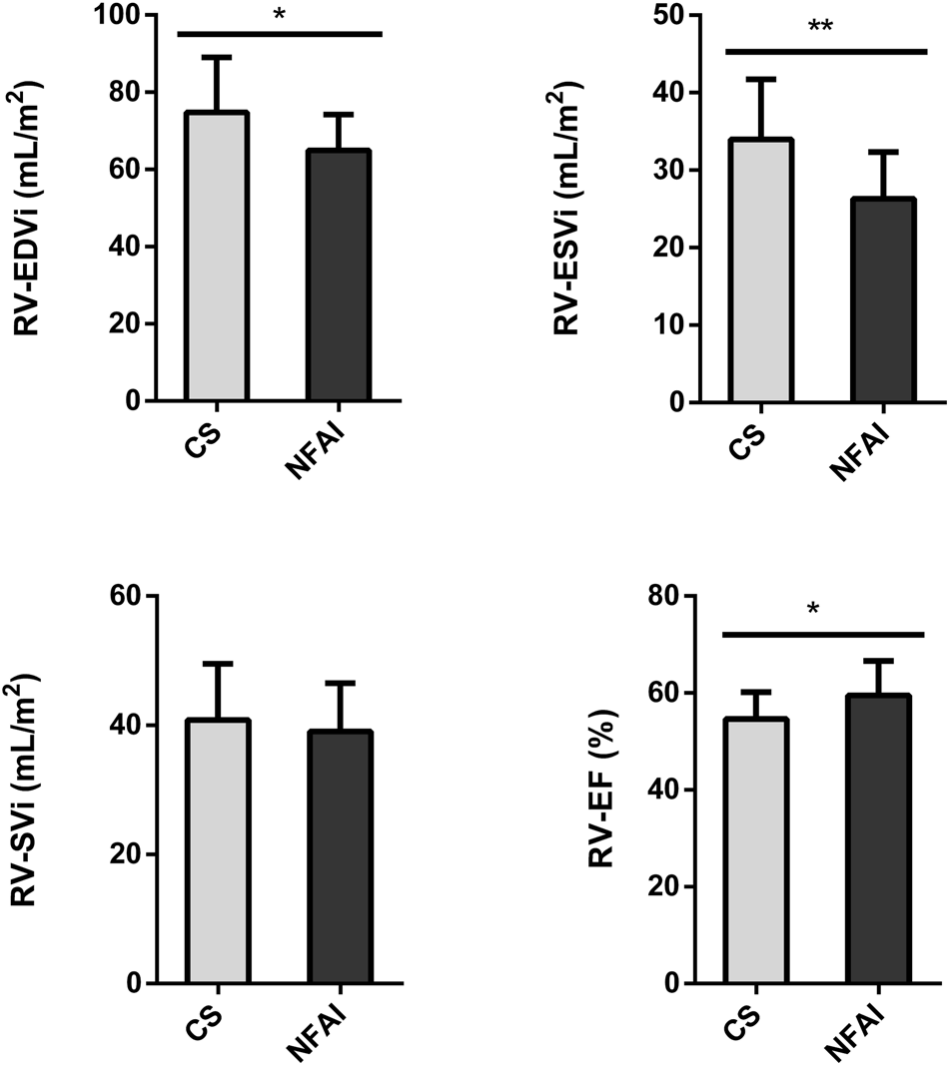
Comparative analysis of right ventricle parameters in Cushing’s syndrome and NFAI patients. Right ventricle morphological and functional cardiac parameters in patients with Cushing’s syndrome (gray bars) and patients with NFAI (black bars). Data are expressed as mean ± SD. *p < 0.05; **p < 0.01; CS Cushing’s syndrome, CNT NFAI, RV-EDVi Right Ventricle End-Diastolic Volume index, RV-ESVi Right Ventricle End-Systolic Volume index, RV-SVi Right Ventricle Stroke Volume index, RV-EF Right Ventricle Ejection Fraction

A comparison of the main morphostructural and functional cardiac parameters between CS and NFAI in the left and the right ventricle is reported in Fig. 2 and Fig. 3, respectively. CMR cardiac morphology revealed an increased left ventricle-end systolic volume index (LV-ESVi) (31.0 ± 8.7 vs 24.1 ± 7.4, *p* = *0.027*) in CS compared to NFAI (Fig. 2). Left ventricle mass index (LVMi) was also higher in CS (51.0 ± 11.8 vs 41.8 ± 6.9, *p* = *0.013*) (Fig. 2), albeit none matched the criteria for left ventricular hypertrophy [37]. Regarding cardiac function, a trend toward lower left ventricle-ejection fraction (LV-EF) was measured in CS (57.1 ± 6.3 vs 61.9 ± 7.0, *p* = *0.056*). Mirroring the alterations found in the left ventricle, higher indexed right ventricle-end systolic volume (RV-ESVi) (34.0 ± 7.7 vs 26.3 ± 6.0, *p* = 0.006) and right ventricle-end diastolic volume (RV-EDVi) (74.8 ± 14.2 vs 64.9 ± 9.3, *p* = *0.035*), as well as lower right ventricle-ejection fraction (RV-EF) (54.6 ± 5.5 vs 59.5 ± 7.1, *p* = *0.044*) were measured in patients with CS (Fig. 3). Dedicated T1 mapping technique did not reveal any difference between CS and NFAI, either before or after contrast administration. No patient had ECV greater than 30%, and no difference in ECV values was observed between groups.

No significant correlations were found between CMR parameters and UFC x ULN (assessed at diagnosis or date of CMR evaluation), serum cortisol after dexamethasone suppression test (at diagnosis) or disease duration from diagnosis. An explicative summary of cardiac parameters is shown in Table 3.

**Table 3:** Cardiac parameters in CS patients and NFAI

	CS (*n* = 16)	NFAI (*n* = 15)	*p* value
LV-EDVi (ml/m^2^)	71.2 ± 15.1	62.9 ± 11.8	0.099
LV-ESVi (ml/m^2^)	31.0 ± 8.7	24.1 ± 7.4	**0.027**
LV-SVi (ml/m^2^)	40.3 ± 8.0	38.7 ± 7.1	0.565
LV-EF (%)	57.1 ± 6.3	61.9 ± 7.0	0.056
LVMi (g/m^2^)	51.0 ± 11.8	41.8 ± 6.9	**0.013**
Concentricity index (g/mL)	0.72 ± 0.14	0.71 ± 0.18	0.803
IVS thickness (mm)	10.0 (8.2–12.7)	10.0 (8.0–10.0)	0.572
RV-EDVi (ml/m^2^)	74.8 ± 14.2	64.9 ± 9.3	**0.035**
RV-ESVi (ml/m^2^)	34.0 ± 7.7	26.3 ± 6.0	**0.006**
RV-SVi(ml/m^2^)	40.8 ± 8.7	39.0 ± 7.5	0.554
RV-EF (%)	54.6 ± 5.5	59.5 ± 7.1	**0.044**
T1-preMean (ms)	1003.4 ± 25.0	1001.1 ± 18.2	0.834
T1-postMean (ms)	443.0 ± 60.5	413.1 ± 42.1	0.133
ECV (%)	25.1 ± 2.3	25.9 ± 2.2	0.320

Values are presented as mean ± SD or as median, CI [5–95] according to the distribution of variables. Bold values indicate a *p* value lower than 0.05 as statistically significant

*P* values < 0.05 were considered statistically significant

*CS* Cushing’s syndrome, *NFAI* non-functioning adrenal incidentalomas, *n* number, *LV* left ventricle, *EDV* end-diastolic volume, *EDVi* end-diastolic volume index, *ESV* end-systolic volume, *ESVi* end-systolic volume index, *SV* stroke volume, *SVi* stroke volume index, *EF* ejection fraction, *LVM* left vetricular mass, *LVMi* left vetricular mass index, *LVH* left ventricular hypertrophy, *IVS* interventricular sept, *RV* right ventricle, *ECV* extracellular volume

## Discussion

The current study reveals that exposure to endogenous GC excess induces a peculiar early remodeling of affected patients’ left and right ventricles, which can persist after CS remission and is independent of traditional cardiometabolic risk factors. Namely, the higher LV and RV ESVi and EDVi observed in patients exposed to GC excess is accompanied by higher LVMi. However, LV and RV ejection fractions are only mildly reduced, suggesting that a morphological impairment anticipates a performance dysfunction. The fact that such alterations occur rapidly in CS and are partially irreversible after remission advocates the use of CMR to improve the management of fatal cardiac complications in this rare endocrine disease.

Several echocardiographic studies have evaluated cardiac structure and function in patients with CS and found LV systolic and diastolic dysfunction [19, 21, 38–41]. Albeit cardiac echocardiography is more practical in everyday clinical practice, CMR allows an evaluation of ventricular mass and volumes free of cardiac geometric assumption, ensuring a higher accuracy and reproducibility [29, 42].

Few controlled studies evaluating small cohorts have analyzed patients with CS using CMR [18, 26–28]. Kamenicky and coworkers compared 18 patients with active CS with 18 controls matched for age, sex, and BMI and found that patients had lower LV, RV, and left atrium ejection fractions, along with increased left and right ESVi and end-diastolic LV segmental thickness. Of note, successful treatment of CS was associated with an improvement in ventricular and atrial systolic performance [18]. A later study from the same group evaluated 23 patients with active CS and compared them with 27 controls matched for age, sex, and BMI, reporting increased left ventricular wall thickness, and reduced ventricular stroke volumes in patients [28]. A CMR study comparing CS patients with age and sex-matched controls showed that patients with active disease had higher LVMi than controls, as opposed to those in disease remission [27]. In all the studies mentioned above, patients and controls significantly differed in cardiovascular risk factors, with a worse cardiovascular profile in patients than controls. Conversely, our cohorts were largely homogeneous, without any significant difference between patients with CS and NFAI in glycometabolic profile, except for a surprisingly marginally lower fasting glucose levels in CS than in NFAI. This is likely because CS patients were either cured or drug-treated, and NFAI were comparable in terms of BMI and known CV risk factors.

As a result, the two groups did not significantly differ either in systolic and diastolic blood pressure levels or in the overall prevalence of cardiometabolic complications or the drugs prescribed to treat them. Nevertheless, our results confirmed the CMR findings of previous studies regarding higher cardiac volumes and mass and lower ejection fractions in patients with CS than in NFAI, advocating a direct effect of GC excess exposure in cardiac impairment beyond the known cardiovascular risk factors. Moreover, the results of the current study highlight the importance of a biventricular evaluation in this context, as opposed to most 2D-echocardiographic studies. Ultrasound measurement of RV volumes is challenging; therefore, most CS echocardiographic studies have mainly focused on the LV [19, 21, 38–41], whereas CMR studies suggest an impairment in both left and right ventricles. The RV is anatomically and functionally different from the LV. In the absence of clear alterations in pulmonary resistance, our findings suggest RV involvement is a direct effect of GC excess on cardiomyocytes, whose receptors are equally expressed in left and right ventricles in donor hearts and dilated cardiomyopathy [43].

Cardiac morphological alterations in our cohort were not related to increased myocardial fibrosis, as we did not find any difference between patients and controls in T1 mapping evaluation, probably also due to the superimposable cardiometabolic profile of the two study groups. Albeit patients had non-significantly higher postcontrast T1 values, none had ECV values compatible with fibrosis. Similarly, Roux and coworkers evaluated 10 patients with active CD matched with 10 hypertensive and 10 healthy controls and performed a CMR study using the T1 mapping technique and found increased native myocardial T1 in CD, independently from hypertension, without differences in myocardial partition coefficient (λ) between groups. These results support the hypothesis of a potential role of T1 mapping in identifying early biomarkers of subclinical myocardial fibrosis in this disease [26].

Even though we didn’t find any significant correlation between indicators of hypercortisolism severity (UFC x ULN, disease duration) and CMR parameters, the independency of cardiac alterations from traditional cardiometabolic risk factors, claims a direct role of hypercortisolism on cardiac impairment, acting as a fingerprint of GC excess exposure. Our data point toward a persistent toxic effect on the heart, mediated directly through GC and/or mineralocorticoid receptors [2, 11, 44], that produces changes in cardiac structure that are clinically silent but long-lasting, as if the heart retained a memory of GC excess exposure. The mineralocorticoid pathway increases collagen secretion by activating fibroblasts [45]. In addition, stimulating mineralocorticoid receptors decreases myocyte contractility and stimulates mitosis, resulting in myocardial hypertrophy and dysfunction [46]. However, previous data on mineralocorticoid antagonism in GC-induced hypertension did not prove convincing [47], disclosing the need for direct control of GC receptors (for example, via selective GC receptor antagonists such as relacorilant). Indeed, there is evidence supporting the role of GCs in driving alterations in vasoactive substances, thus impacting the balance between vasoconstriction and vasodilation (including catecholamines, nitric oxide, and atrial natriuretic peptide), as well as the activation of the renin-angiotensin system, leading to cardiac hypercontractility [11, 48].

The present study has shown more structural rather than functional changes at CMR in CS patients without evidence of fibrosis, thus suggesting the latter probably as a late phenomenon.

Additive to the direct role of cortisol, almost 60% of our patients presented hypertension, which could have contributed to the development of cardiac impairment. Similarly, the impact of other CS-related cardiovascular risk factors, such as visceral obesity, glucose intolerance and dyslipidemia, cannot be entirely ruled out.

Finally, our study showed for the first time that sex might affect cardiac morphological changes induced by GC excess. Male CS patients exhibited higher IVS thickness compared to females after adjusting for population age and sex reference ranges, independently from the prevalence of hypertension, supporting sex-related differences as observed in other cardiovascular diseases [49, 50]. Recently, a study by Wolf et al. showed that male sex was an independent predictor of increased epicardial and pericardial fat [28], which may play a role in the pathogenesis of CS cardiomyopathy. However, among CS patients, we did not find any differences in the prevalence of male and female hypogonadism (50% vs 42%, *p* = 1.000). Still, we can not exclude that estrogen exposure could have protected GC-related cardiomyopathy [51].

Very few studies have evaluated cardiac structure and function in CS using CMR, and this is a strength of the current study. However, it does have some limitations. The cross-sectional design and the lack of sample size in such a small and heterogeneous study population with different etiologies of endogenous CS, including both cured and well-controlled patients, might have underestimated the cardiac impairment. Anyway, considering that CS is a rare disease, we opted for a study design closer to a CS clinic’s real-life setting; this aspect represents a strength of this study. Nevertheless, according to the published evidence, as well as to our results, it is likely that cardiac dysfunction might persist in CS even after disease remission. Indeed, although our CS patients had higher biventricular volumes, the subgroup comparison between surgically cured and drug-treated patients revealed no differences in cardiac morphology or biochemical or cardiometabolic complications prevalence, althoghut the lack of standardization of the evaluation period. A previous paper found a significantly higher LVMi in active patients than in remission [27]. Anyway, longitudinal studies (baseline versus post-treatment) with larger population are needed to better clarify the reversibility of cardiac changes after treatment.

We propose a novel approach to cardiac disease in CS, going beyond the traditional cardiometabolic risk factors and evaluating both ventricles, preferably with CMR. Moreover, the present study highlights the importance of a sex-oriented approach in the management of CS complications, taking into account the sex-related differences in cardiac damage of these patients, for whom cardiac complications still represent the major cause of death, very often occurring during remission [2].

## Conclusions

In CS biventricular cardiac remodeling associated with functional impairment, has been ascribed to a multifactorial pathogenesis. Our findings highlight the greater contribution of direct effect of GC excess exposure on myocardium than on cardiovascular risk factors, suggesting a sex-related differences in cardiac impairment. More importantly, the maladaptive change triggered by chronic exposure to GC excess, even if the latter is resolved, is persistent and clinically silent and could be detected though a more sensitive and precise approach with CMR.

## Data Availability

The datasets used and/or analyzed during the current study are available from the corresponding author upon reasonable request.
